# The International Medical Congress

**Published:** 1867-11

**Authors:** E. Andrews

**Affiliations:** Paris


### THE INTERNATIONAL MEDICAL CONGRESS.

PARIS, Aug. 17, 1867.

This body of medical men, selected from the whole civilized world—from San Francisco to Constantinople, at least—assembled yesterday for the first session. The meetings are held in the Ecole de Medicine, the great school of the Paris Faculty of Medicine. In some respects, the French are behind the age in conducting voluntary conventions. There has been very little pains taken to render the advent and the stay of delegates agreeable and convenient. All that free-hearted zeal which welcomes our American Medical Association to its yearly gathering, and strives, in numberless ways, to make the members enjoy their visit, is wanting here. The Parisians seem to suppose that it is a sufficient blessing to outside barbarians to be permitted to come to the Parisian Paradise, and hear the medical angels expound their discoveries. The *concierge* at the gate did not know where the Secretary was, when he would be in, nor whether the Congress was to have such an officer, until numerous inquiries awakened a suspicion of the fact in his mind. No directions were posted, as to where credentials were to be presented, or names recorded. The delegates ran about the building in a bewildered state, inquiring of each other where the Secretary was, using first one language, and then another, as if a man who did not know a fact in French or English, might possibly be aware of it in Dutch. Finally, the Secretary was discovered in a side apartment, with no clue to its location visible. He looks at the credentials, but keeps no general register or directory in a visible form for the reference of delegates. Probably, he has a list somewhere, for the records.

The sessions are held, as I said before, in the Ecole de Medicine. This is, in some respects, a fine building, but characteristic of the French nation in its arrangements. It has an expensive and imposing colonnade in front, consisting of some thirty or forty pillars of hewn stone. The amphitheatre has a vaulted ceiling, elaborately panneled and ornamented, and the walls over the desk are hung with some immense and costly paintings. The walls are draped with velvet and damask around the entire room, and above hang the flags of all nations, in graceful clusters. The seats—what a descent!—the seats are benches of bare boards, ten inches wide, and without any backs. The French students who sit for seven years on such seats, must have the patience of martyrs. I recommend the government to sell one or two of the stone columns in front, and appropriate the proceeds to the construction of some comfortable chairs for the pupils.

At 2 P.M., August 16, the President, Prof. Bouillaud, who is renowned for having first brought to light the connection between rheumatism and carditis, entered the desk, and called the assembly to order. About 700 were present. The Secretary-General, Dr. Jaccoud, assisted him, and by his great power and clearness of voice in all his announcements and readings, made atonement for the obscurity of his whereabouts before. The President then read a lively and brief address, which showed that he had gathered neither loquacity nor dulness, by his advanced age. He welcomed the delegates to Paris, and pointing to the standards of the various nations, grouped around the walls, he said: "Let us salute these flags, then, by uniting our hands, as these interlaced banners are united, in sign of complete cordiality and entire fraternity." The gracefulness of the figure took the audience by surprise and brought down a storm of applause.

After the close of the address, the reading of the papers commenced. The topic of the day was expressed as follows: "*Anatomy and Physiology of Tubercle. Tuberculization in the Different Countries, and its Influence upon the General Mortality.*" The papers presented, were by Prof. SANGALLI, of Pavia; Dr. VILLEMIN, of Paris; Prof. CROCQ, of Brussels; Prof. LEBERT, of Breslau; Dr. MARMISSE, of Bordeaux; Dr. D. LEE, of London; Dr. LARRAMEA, of Bordeaux; Dr. ULLERSPERGER, of Munich; Dr. HOMAN, of Christiana.

After two papers had been read, it became evident that the session was likely to last many hours, and the audience became restless.

Dr. VAN LOHE, a Hollander, rose and began to express his dislike of the order of business and his desire that the Congress should use its rights, and discuss such topics as it deemed best, instead of simply hearing a series of papers read. He commenced as follows:

"Mr. President, is it allowable for me to ask a question?"

The President said: "Certainly, proceed."

He proceeded, therefore, in this wise: "I am a foreigner; I am a Hollander, and, as a Hollander, was sent into this assembly to assist at the Congress. But, up to the present time, I find myself mistaken. It looks to me as if this were not an International Medical Congress, but a court, or a college where the physicians concerned have got together to hear each other read, and for mutual admiration."

The audience waked up at this thunderbolt, and began to applaud the bold Hollander. The President, however, checked him, and courteously reminded him that there was a regular programme of topics, to which it was necessary to conform. Dr. V. L., however, was not to be put down, and proceeded with his speech. The President tried, in vain, to stop him. Finally, the audience deemed that he was going too far, and hissed him down. He took his hat and went out. He reappeared, however, the next day, and the President offered him an opportunity to finish his remarks, but he did not choose to avail himself of the permission.

August 17, at 8 P.M., the second session commenced, there having been no meeting during the day.

The first paper read was on a new operation for opening abscesses of the liver, which had been practiced in Mexico. The second was by Dr. GALEZOWSKI, of Paris, on the alterations of the retina in the tuberculous diathesis. Some fine colored drawings were shown, displaying the increased vascularity of the retina in tubercular meningitis, as seen through the ophthalmoscope.

Another paper was read on the same topic, the two together being illustrated with some forty colored drawings of diseased retinæ. Two papers were also read on the treatment of tubercle. After each reading, time was allowed for discussion, which was embraced with more ease and skill than is usual in the American Medical Association. Each speaker advanced to the tribune, and addretsed the Congress in a seated posture, a rather awkward position for a speaker; however, the remarks were brief, vigorous, and lively. There was less stiffness than in American scientific bodies, wit flowed freely, and the Congress laughed with great hilarity.

I send this report by the steamer about to sail, and will forward the remaining proceedings by a future mail.

E. ANDREWS, M.D.

